# Prediction of Neonatal Respiratory Distress Biomarker Concentration by Application of Machine Learning to Mid-Infrared Spectra

**DOI:** 10.3390/s22051744

**Published:** 2022-02-23

**Authors:** Waseem Ahmed, Aneesh Vincent Veluthandath, David J. Rowe, Jens Madsen, Howard W. Clark, Anthony D. Postle, James S. Wilkinson, Ganapathy Senthil Murugan

**Affiliations:** 1 Optoelectronics Research Centre, University of Southampton, Southampton SO17 1BJ, UK; waseem.ahmed@soton.ac.uk (W.A.); avv1a15@soton.ac.uk (A.V.V.); d.rowe@soton.ac.uk (D.J.R.); jsw@ecs.soton.ac.uk (J.S.W.); 2 Neonatology, Faculty of Population Health Sciences, EGA Institute for Women’s Health, University College London, London WC1E 6AU, UK; jens.madsen@ucl.ac.uk (J.M.); h.clark@ucl.ac.uk (H.W.C.); 3 Academic Unit of Clinical & Experimental Sciences, Faculty of Medicine, Southampton General Hospital, Southampton SO16 6YD, UK; adp@soton.ac.uk

**Keywords:** ATR–FTIR, spectroscopy, machine learning, neonatal respiratory distress syndrome, point-of-care devices

## Abstract

The authors of this study developed the use of attenuated total reflectance Fourier transform infrared spectroscopy (ATR–FTIR) combined with machine learning as a point-of-care (POC) diagnostic platform, considering neonatal respiratory distress syndrome (nRDS), for which no POC currently exists, as an example. nRDS can be diagnosed by a ratio of less than 2.2 of two nRDS biomarkers, lecithin and sphingomyelin (L/S ratio), and in this study, ATR–FTIR spectra were recorded from L/S ratios of between 1.0 and 3.4, which were generated using purified reagents. The calibration of principal component (PCR) and partial least squares (PLSR) regression models was performed using 155 raw baselined and second derivative spectra prior to predicting the concentration of a further 104 spectra. A three-factor PLSR model of second derivative spectra best predicted L/S ratios across the full range (*R*^2^: 0.967; *MSE*: 0.014). The L/S ratios from 1.0 to 3.4 were predicted with a prediction interval of +0.29, −0.37 when using a second derivative spectra PLSR model and had a mean prediction interval of +0.26, −0.34 around the L/S 2.2 region. These results support the validity of combining ATR–FTIR with machine learning to develop a point-of-care device for detecting and quantifying any biomarker with an interpretable mid-infrared spectrum.

## 1. Introduction

Point-of-care testing presents an opportunity to improve clinical care by facilitating rapid access to appropriate patient management and delivering a higher standard of patient care. Such testing of clinical samples can provide rapid evidence-based insights into patient conditions and, particularly in emergency cases and where conventional laboratory-based tests are time-consuming, the rapid availability of clinically relevant information can have a direct impact on patient outcomes.

Point-of-care (POC) testing requires, amongst other factors, an understanding of disease pathology, the identification of specific biomarkers, near-patient administration, and a compact form-factor consistent with ‘bedside’ use [1]. Mid-infrared (mid-IR) spectroscopy offers one potential solution for such a scenario and provides a sound basis upon which to base POC devices [2]. Using spectroscopic techniques to analyze the molecular vibrations of an applied bio-sample, the functional groups and fingerprints of the constituent chemicals can be not only identified but also quantified. This approach avoids the requirement for any labelling protocols, and rapid diagnostic devices can provide ‘real-time’ measures of patient conditions and be packaged in a desktop format.

## 2. Background

Clinical outcomes for the treatment of patients depend on the ability of a clinician to understand the conditions underlying the presented symptoms. When diagnostic information is limited, clinicians must rely on experience and skill and treat patients cautiously. Early intervention in many conditions can either prevent or decrease disease severity [3,4], but only a sub-set of patients often respond to treatment. Consequently, in the absence of diagnostic information to identify which patients would benefit from treatment, inappropriate or unnecessary intervention can lead to over-treatment with potentially harmful side effects. Neonatal respiratory distress syndrome (nRDS) is one condition that presents clinicians with such a dilemma; in this condition, preterm infants (born 10–15 weeks early) can develop nRDS as a consequence of lung immaturity [5] and a lack of lung surfactant. Surfactant reduces surface tension forces [6] within the alveoli, is essential for gas exchange [7], and facilitates the transition from fetal to post-natal life. The fundamental cause of nRDS is alveolar collapse due to high surface tension, with preterm neonates presenting as cyanotic and tachypneic, along with audible grunting and a characteristic ground glass appearance on X-ray [8]. Treatment for nRDS involves mechanical or non-invasive ventilation combined with increased inspired oxygen concentration [9]. However, though such treatments are essential to prevent death, the combination of barotrauma and oxidative damage can themselves cause long-term lung damage and the development of neonatal chronic lung disease (nCLD) [10,11]. Therapeutic administration with exogenous surfactant soon after birth has proven very successful in treating nRDS [12,13] and decreasing the incidence of nCLD, but not all preterm infants develop nRDS. There are no established diagnostic tests within a clinically relevant time frame to predict which infants will develop nRDS and benefit from surfactant therapy. In our current study, we posit the use of attenuated total reflectance (ATR) Fourier transform infrared (FTIR) spectroscopy as a viable method to perform such analyses and provide clinically relevant information at the point of care, consistent with a bedside diagnostic platform.

### 2.1. Diagnosis of nRDS

As nRDS is related to lung immaturity, efforts to produce a clinical test have focused on biomarkers present within samples drawn from amniotic fluid (AF) [14], in lung lavage (bronchoalveolar lavage (BAL)) [15], and the fluid found in the neonate stomach soon after birth (gastric aspirate (GA)) [16]. The analysis of the lecithin/sphingomyelin ratio (L/S ratio) in AF was established by Gluck [17,18] and shown to correlate with lung maturity, but the thin layer chromatography (TLC) method employed in the study was laborious and required considerable skill rendering it unsuitable for point-of-care testing. Since then, further attempts have been made to measure the L/S ratio using mass spectrometry [19] and transmission infrared spectroscopy [20], but all methods still require significant equipment or time, making them unamenable to point-of-care diagnostics.

The lecithin (L) component of surfactant increases in concentration with gestational age compared to sphingomyelin (S or SM), which remains relatively constant throughout and acts as an internal standard for this measurement. The largest constituent of L is dipalmitoylphosphatidylcholine (DPPC) (approximately 40% [21]). The reported amounts of total recovered phospholipids from BAL are in the range from 0.15 to 0.88 μmol, which corresponds to between 0.12 and 0.6 μmol/m of BAL [15]. The actual molar concentration can be adjusted by varying the amount of trichloromethane used for the extraction of the lipids from a patient sample. Similarly, recovering a larger volume of patient sample in the extraction process increases the quantity of sample available for testing. L/S ratio samples used for analysis with TLC [22,23] have traditionally been prepared by centrifugation followed by liquid–liquid extraction [24,25] to separate the lipid portion (found in the lowest layer) from any other contaminants using trichloromethane, methanol and water. Verder et al. [26] followed a similar method to prepare samples for mass spectrometry but using dichloromethane instead of trichloromethane to contain the lipid fraction, avoiding the human-health-related safety issues associated with the use of trichloromethane. The advantage of such an extraction technique is that it removes other interfering components of AF/BAL/GA, such as proteins, leaving only the lipid component containing the phospholipids biomarkers that can be further analyzed.

### 2.2. Machine Learning for Spectral Analysis

Using a univariate approach for the analysis of ATR–FTIR spectra requires that a single peak corresponding to the absorbance from DPPC is compared to a single peak corresponding only to SM absorbance to establish the L/S ratio of any given sample. Recorded information other than these peaks, which could otherwise be used to train the model, are disregarded, thus wasting collected data. Multiple linear regression is the multivariate extension of linear regression and uses multiple peaks to model spectra; it is limited by the number of wavelengths for which data are recorded. To build a model that can account for such a high number of data dimensions, one must perform a number of spectral scans exceeding the number of variables. Such an approach is difficult to achieve in practice.

Multivariate linear regression assumes that each variable is independent of another, which is not the case for spectroscopic data. Using techniques such as principal component analysis can reduce the dimensions of data by fitting each successive principal component onto orthogonal directions of greatest variance. This overcomes the limitations of multiple linear regression and ensures that enough degrees of freedom are retained within the experiment. Further improvements can be obtained using the known concentrations of a calibration set to maximize the covariance between the spectral data and the concentrations, such as is done when performing partial least squares regression (PLSR). 

It is important that the calibration of the models is performed using representative samples (ideally patient samples) to include all the absorbing species present, along with their associated variability. This variability is not correlated with the target analyte concentration, thus allowing the machine learning algorithms to model the regions of the spectra associated with the required concentration changes.

A crucial aspect in providing measurements in a clinical context [27] is the provision of an indication of the associated uncertainty to convey how much confidence clinicians should have in the figures returned by any diagnostic aid. When simple models such as linear regression are used and the assumptions of normality for the model outputs hold, then providing prediction intervals is a straightforward endeavor. However, when the normality assumption does not hold, non-parametric analyses to generate appropriate prediction intervals must be used. Bootstrapping is one method with which such prediction intervals can be built [28], can be applied to both parametric and non-parametric model outputs, and can be used to compare the performance of models on the basis that a narrower prediction band for a given confidence level has less associated uncertainty.

### 2.3. Use of ATR–FTIR to Measure the L/S Ratio

There has been much interest in the use of mid-IR spectroscopy in a clinical context [29,30,31] because it fulfils the requirements of the POC paradigm, in that it can be used to perform quantitative rapid, label-free measurements of a patient sample. However, the presentation of the sample and the interpretation of the gathered data are both topics that would benefit from further development. Ideally, samples would require no preparation prior to measurement, and the interpretation could be directly understood by a non-specialist clinical end user. L/S ratios from GA samples have already been measured by ATR–FTIR using dried samples with a BioATR^®^ Cell accessory [26], and the results were useful in establishing that ATR–FTIR is an appropriate tool for measuring the L/S ratio. However the cleaning of the ATR crystal between tests still presents problems; ATR crystals are too expensive to be disposable and significant effort must be expended to clean them between tests. Testing with liquid samples presents an easier proposition for cleaning and extends the method for use with any other liquid biomarker [32]. Therefore, in this study, we used biomarkers in the liquid form.

In addition, drying analyte samples on an ATR crystal poses some challenges for spectrometric analysis. First, the process of drying has the potential to cause the separation of analyte components [33,34], which means that the sample is no longer homogenous above the ATR crystal. In a worst-case scenario, this could cause locally high concentrations of the analyte of interest, resulting in the inference of false diagnostic information. This would be especially important to consider when, in a drive to realize smaller diagnostic devices based on ATR and excited by tunable IR lasers, such differences may hinder the collection of representative spectra depending on which portions of the analyte are illuminated by the laser beam.

The thickness of films produced by drying can have an impact on the spectra collected [35], showing larger absorbances for thicker films—the effect being particularly more pronounced at longer wavelengths (lower wavenumber). For the quantification of analyte concentration from the spectra alone, this represents an additional source of error that needs to be addressed during calibration. This effect must also be considered in the background of ATR spectra, which generally display a larger absorbance at longer wavelengths due to the longer optical path these wavelengths traverse within the analyte medium when compared to normal-incidence transmission spectra. Cracks in dried samples lead to scattering-related loss, adding further uncertainty to the absorption peak intensity, requiring extra pre-processing, and complicating the analysis.

The authors of the current study generated L/S ratios using synthetic DPPC and SM dissolved in a suitable solvent and performed ATR–FTIR measurements on them in the liquid phase, thereby overcoming the issues present when measuring dried samples. The generated spectra were used to train machine learning models that further predicted the concentrations of each species present, and these concentrations were used to estimate the L/S ratios of the applied samples based on the spectrum alone.

## 3. Methods

The SM (Avanti Polar Lipids) and DPPC (Merck) were used as purchased in powder form without further processing. SM is a sphingolipid formed from a sphingosine backbone with a 16-carbon acyl group attached by an amide bond and a phosphatidylcholine headgroup. DPPC is a glycerophospholipid based on a triglyceride backbone with two saturated 16 carbon acyl chains attached by an ester linkage and a phosphatidylcholine headgroup. To analyze in a liquid medium, it was necessary to identify a suitable solvent for both SM and DPPC, which has a mid-IR spectrum that does not significantly overlap with that of SM and DPPC and has evidence of prior use. The samples in this case were purified versions of the same reagents found in lung surfactant, thus presenting an ideal set of data to train the machine learning models. Developing machine learning models on these data therefore set a baseline against which subsequent, more complex, data could be analyzed. Further investigations can apply the same analytical methodology to patient samples with the aim to optimize the full workflow from collecting the patient sample up to presenting the data to an end user.

### 3.1. Solvent Selection

L/S ratio samples for analysis by TLC [22,23] have traditionally been prepared by centrifugation followed by liquid–liquid extraction [24,25] to separate the lipid portion (found in the lowest layer) from any other contaminants using trichloromethane, methanol and water. Verder et al. [26] followed a similar method to prepare samples for mass spectrometry, though they used dichloromethane instead of trichloromethane to contain the lipid fraction, avoiding the human-health-related safety issues associated with the use of trichloromethane. The advantage of such an extraction technique is that it removes other interfering components of AF/BAL/GA, such as proteins, leaving only the lipid component containing the phospholipids biomarkers. In this study, we therefore adopted dichloromethane as the solvent of choice, notwithstanding the fact that there are regions in which dichloromethane and the phospholipids share absorbances. Compared to the mid-IR spectrum for trichloromethane, the spectrum for dichloromethane shows less strong absorbances overall, thus allowing for the finer spectral features of the lipids to be elucidated. So that particularly strong absorbances from dichloromethane did not feature in the spectral measurements of the lipids, the region between 1190 and 1310 cm^−1^ was removed (zapped).

### 3.2. Mid IR Spectrometry

Measurements were performed with an Agilent Cary 670 FTIR instrument equipped with a potassium bromide (KBr) beam splitter and a deuterated triglycine sulphate (DTGS) detector. This was controlled by Resolutions Pro software. A 10-bounce Pike^®^ zinc selenide (ZnSe) horizontal ATR accessory with a solvent lid was used as the sample platform. Nitrogen purging was set to 4 L/min. Scan settings were set to 32 readings at a 4 cm^−1^ resolution. The SM and DPPC were used to make stock solutions from which the test solutions were generated. For each measurement, 0.5 mL of analyte was used in the ATR well.

The solutions were prepared by adding measured masses of each component along with the required amounts of dichloromethane. The solutions were heated, if required, for 30 s at 63 °C followed by vortex mixing for another 30 s. This was repeated until the solutions were visibly homogenous and then repeated thrice more. 

It was observed during this process that, when at rest for longer than half an hour, the more concentrated solutions tended to form precipitates in the solution. This observation challenged the assumption that the solutions were homogenous and required that, prior to placing a lipid sample on to the ATR crystal, the solutions were heated 1 min at 63 °C and vortex mixed for 30 s to ensure homogeneity. It was assumed that the temperature of the sample, when placed on an ATR crystal, would rapidly equilibrate with the ambient temperature, leaving the measurement unaffected. This step is not expected to feature in a clinical setting as it is not expected that the samples would be left to stand for any significant length of time (in our experience greater than approximately 30 min). 

The samples were placed in the ATR crystal well using a pipette. It was observed that there was a tendency to form bubbles from within the solution within 10 s of placing the sample. If left for a further 30 s, these bubbles usually disappeared by themselves. This process could be accelerated by the agitation of the solution in the ATR well by the pipette tip. Bubble formation was not observed after this, leading to the belief that this was an artefact of using a pipette to place the sample. Indeed, using a pipette with a smaller tip annulus resulted in less bubble formation. The rate of evaporation of dichloromethane was reduced with the use of a solvent lid to a rate that was insignificant over the length of the measurement.

For each liquid sample (DPPC, SM and L/S) dispensed onto the ATR crystal, between 6 and 18 successive scans were recorded. These included spectra containing artefacts relating to the water vapor difference between the background and sample measurements, ensuring that at least one recorded spectrum was recorded free of water vapor interference. To introduce the variability associated with a clinical-use scenario, a background spectrum was recorded prior to each set of scans, as this was expected to be different for each measurement depending on the performance of the particular FTIR machine used.

### 3.3. Cleaning the ATR Crystal

The cleaning of the ATR crystal was performed in situ by adding 1 mL of dichloromethane and using a cotton bud to agitate the solution present in the ATR well before wicking the solution out using a KimWipe. This process was repeated three times each time the sample in the ATR well was changed. A clean ATR crystal was assessed by recording a spectrum of dichloromethane without performing a new background and observing peaks around 2900 cm^−1^ corresponding to the lipid components with a peak height less than 0.0004 A.U. This level was chosen due to no further reductions being observed after further repeating the cleaning steps. The effect of this on the spectra was mitigated by performing measurements from the smallest to the largest concentration.

### 3.4. Spectral Processing

Once collected, the spectra were subject to processing in EssentialFTIR software (Operant LLC), which truncated the spectra to 830–3500 cm^−1^ and then applied a baseline offset correction to all the spectra at 2500 cm^−1^. Finally, the spectral information in the regions responsible for carbon dioxide absorption (2150–2430 cm^−1^) and the known strongest dichloromethane absorption (1190–1310 cm^−1^) were removed by zero filling and then adding Gaussian noise (mean = 0, standard deviation = 4 × 10^−5^ A.U.). Gaussian noise was added to match the noise standard deviation extracted from the spectra in the region of 2450–2640 cm^−1^, in which no sample absorbances were observed, using the signal-to-noise ratio tool in EssentialFTIR. Further data processing was performed in Python using Jupyter Notebook.

### 3.5. DPPC and SM Spectral Measurement

The mid-IR spectra for both DPPC and SM were recorded to understand the presence of spectral features that could distinguish between them. Both the molecules fall within the category of lipid molecules and share similar functional groups, albeit with slight physical differences that could be observed in the spectra.

### 3.6. L/S Ratio Cut-Off and Sample Generation

The nominal cut-off L/S ratio between disease and non-diseased states was taken to be 2.2 based on the ATR–FTIR study by Verder [26], as those experiments were the most similar to the ones presented in this study. Some studies, using other methods, have posited a cut off at 2.0 [18,19], and others have reported much higher values such as 3.05 [36].

L/S ratio solutions were generated on the basis of a 1 mM SM solution upon which the DPPC ratio could be varied. Solutions were made at L/S ratios of 1.0, 1.7, 2.0, 2.1, 2.2, 2.3, 2.4, 2.7, and 3.4 to provide broad coverage of the values either side of the cut-off while focusing on the ability of the approach to discern ratios close to it, within the so called diagnostic “grey zone” [37].

### 3.7. Chemometric Analysis

The choice of analytical algorithms for this study was based on their ability to use the whole spectrum to train a regression model and the ability to provide quantitative information on the constituent components. The selected algorithms were principal component regression (PCR) and partial least squares regression (PLSR).

Chemometric models require calibration with data taken from samples representative of the target analyte. In order to overcome the effects of interferents or any other factors that did not correlate with the concentration of the target analyte, it was necessary to calibrate the model in a manner that included the effects of these potentially confounding influences. The generated models were only as robust as the information that was provided to them during calibration, and for this reason spectra with water vapor interference present were specifically included. In a clinical setting, such calibrations may also include the presence of other compounds that share absorbance regions, the effect of temperature on the spectra, or even the interaction between the target analytes within a sample. This necessitates that multiple measurements are made in order to model the effect of signal-to-noise ratio at a given concentration. The measurement of small differences in concentration for a calibration set should also be performed using small concentration increments so that the algorithm can discern the changes in the spectra that are due to noise and those correlating with concentration changes. 

To perform the chemometric analysis and assess how likely it was to perform when confronted with new data, it was necessary to split the data into calibration and test datasets. This calibration could be performed using information present within the calibration data alone and the final performance of the model assessed using the test data. This avoided a ‘data leakage’ scenario, in which the model’s performance would be augmented by the test dataset and yield unrealistically good results. In this study, there were 10 groups of data from a total of 259 measurements, with each group corresponding to measurements performed in a single session. The numbers of measurements or ratios tested in each session were not maintained. The groups were randomly split into half, resulting in the allocation of 155 spectra as the calibration set and 104 spectra as the test set.

### 3.8. Model Performance Metrics

The authors of this study chose to assess the ability of the model to fit the data using the *R*^2^ coefficient of determination and mean squared error (MSE). The *R*^2^ indicates how well a regression model fits the known (true) values of the spectra provided, while the *MSE* provides an estimate of the errors within each prediction. A model with a high *R*^2^ and low *MSE* performs better than a model with a low *R*^2^ and high MSE.

For each factor (principal component/latent variable (PC/LV)), each model was generated by taking the calibration dataset and splitting it into a training and cross-validation set (see Supplementary Figure S6 for machine learning workflow). The model was generated using the training data (Tr) and then tested with the cross-validation (CV) data using 10 K-fold cross validations and a random selection of calibration data for each fold. This was performed to an arbitrary number of PCs/LVs greater than that assumed necessary to model most the variance in the data. The performance of each approach in predicting the data in the calibration and cross-validation datasets using the *R*^2^ and MSE was used to assess how many PCs/LVs the final model would contain. A final model was established on the basis that generally using fewer principal components/latent variables was favorable because it generally results in more robust prediction models by avoiding the modelling of noise [38,39].

The models were then retrained using the whole calibration dataset using the indicated number of factors, and their performance was assessed against the test dataset with respect to predicting the concentrations of analytes present in a given sample run and the prediction of the L/S ratio on the basis of these predicted concentrations. The predictions obtained from the models were termed ‘regressed’ or ‘predicted’ values and compared against the known applied (true) values to obtain the *R*^2^ and *MSE* metrics.

Prediction intervals were generated with the bootstrap method [40,41] using the calibration set data. The prediction interval for each point in the test set was obtained for each of the models and provides an indication of the uncertainty associated with each predicted value.

## 4. Results and Discussion

### 4.1. Spectra of DPPC and SM

**Figure 1:**
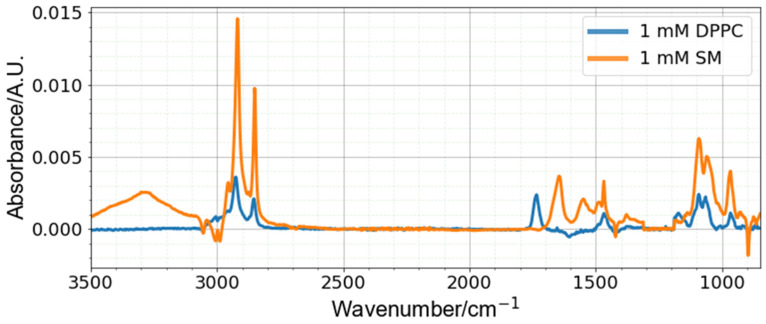
Spectra of DPPC and SM—spectra free of water vapor interference shown.

The recorded spectra from DPPC and SM are shown in Figure 1. The methylene and methyl groups present on the acyl chains for both molecules yielded strong absorbances around 2950 and 2850 cm^−1^, respectively, and the presence of the phosphatidylcholine headgroup provided similar absorbances for both sets of spectra around 1090 cm^−1^. Notable distinguishing features between both sets of spectra include the presence of a peak at 1734 cm^−1^ for DPPC, relating to the ester carbonyl bond, which is absent in the SM spectra. The peak at 1650 cm^−1^ in the SM spectra is related to the presence of amide I bond, which is not present in the DPPC molecule, and similarly missing from the DPPC spectra.

The negative peaks in the spectra (near 3000, 1600, 1420 and 900 cm^−1^) are associated with regions where dichloromethane absorbs in the mid-IR region and are believed to be due to the variation of dichloromethane vapor within the test chamber between the background collection and sample scans. These vapors were gradually removed by the N_2_ purging, and their concentration (peak height magnitude) in the chamber depended on the time taken between the application of the liquid on to the ATR crystal and the actual spectral scan. This was a difficult parameter to control because waiting too long to establish a baseline reading resulted in noisy spectra due to interference from water vapor, but not waiting enough resulted in larger impacts from dichloromethane vapor. Despite this artifact, the machine learning approach performed well for the analysis of L/S ratios from these spectra.

The most significant peaks within each of the DPPC and SM spectra were identified and assigned to the bond vibrations in Table 1. Some of the peaks showed absorbances in similar regions for both DPPC and SM, separated by only a few wavenumbers. Such spectra present a challenge for analysis, particularly in discerning small concentration differences, and require the adopted chemometric approach.

### 4.2. L/S Ratio Spectra

**Figure 2:**
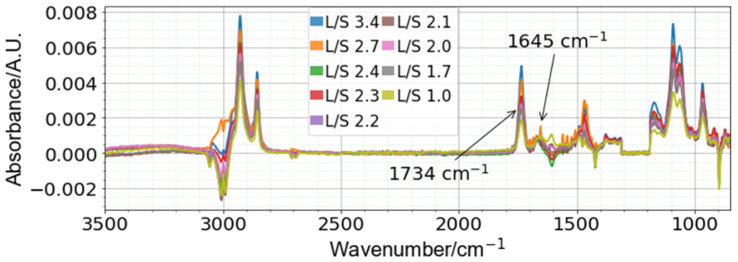
L/S ratio spectra (sample spectra with minimal water vapor present shown) performed at 1 mM SM. The peaks corresponding to 1734 and 1645 cm^−1^ correspond to unique DPPC and SM peaks, respectively.

The spectra of different L/S ratios (Figure 2) show how the spectra changed with increasing DPPC concentration, with the notable increase in the peak at −1734 cm^−1^ (corresponding to the ester carbonyl bond present on DPPC), while the peak at −1645 cm^−1^ (corresponding to the amide I bond present on SM) remained relatively constant. For a univariate approach, comparing these two peaks would be one way to model the concentrations of each DPPC and SM present within an unknown sample.

### 4.3. Spectral Pre-Processing 

The processing of the data was performed with an idea of the likely usage scenario for assessing nRDS in clinical practice such that all information relating to quantification of DPPC and SM was preserved. Complex pre-processing steps that carefully curate each spectrum, while possible in a lab scenario, require that appropriate training is provided to the clinical end user. It is unrealistic to expect that such skills will be available in a point-of-care scenario, so generic pre-processing steps that do not depend on specific spectral features or any user interaction to be present are favored.

The machine learning models used here were generated using the original spectra after baseline correction in EssentialFTIR, as detailed in ‘Spectral Processing’ (termed the ‘original’ dataset), and second derivative of the spectra (termed the ‘second derivative’ dataset), and the impact on the models of this processing step was evaluated. The second derivative spectra had the advantage of removing any additive and multiplicative effects from the data while retaining conformity with the Beer–Lambert law. We also traded an increase in spectral linewidth resolution for a decrease in the signal-to-noise ratio, but this was offset by applying a Savitzky–Golay filter to smooth the result (polynomial order of 3 with a 15-point window) [42].

**Figure 3:**
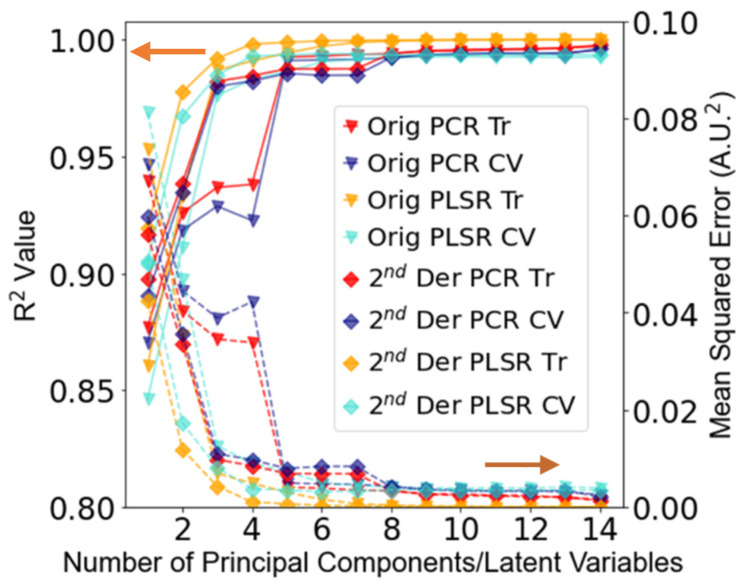
*R*^2^ and *MSE* values for PCR and PLSR models using the training (Tr) and cross-validation (CV) data from the calibration dataset. Solid lines pertain to *R*^2^, and dotted lines pertain to *MSE*.

Since a point-of-care device is likely to be used in scenarios where the effect of water vapor is likely to be a problem, the calibration and test datasets retained all such spectra. Figure 3 shows the performance of the PCR and PLSR models for an increasing number of factors up to an arbitrary maximum of 14, based on the original and second derivative spectra. The best performance on the calibration data was observed with the PCR model calibrated on the original spectra (PCR Orig model) (*R*^2^ *CV*: 0.989, *MSE CV*: 0.006), which modelled the greatest variance in the data. However, since five PCs were required to reach this level of performance, it was expected that this would not perform as well when predicting the test set. The PLSR model applied to the second derivative (PLSR 2D model) spectra performed the next best of the models tested, with more than 3 LVs (*R*^2^ *CV*: 0.985, *MSE CV*: 0.008), showing a slightly further increase in *R*^2^ and decrease in *MSE*. Since this uses less LVs than the PCR Orig model, it was expected that this would generalize better when assessed against the test set. The PCR model calibrated using the second derivative data (PCR 2D model) was also modelled using three PCs (*R*^2^ *CV*: 0.980, *MSE*: 0.010) and outperformed the PLSR model calibrated using the original spectra (PLSR Orig model), which modelled the least variance in the data (*R*^2^ *CV*: 0.976, *MSE*: 0.013) of the models tested.

### 4.4. DPPC and SM Concentration Prediction

The performance of the selected models (Table 2) was assessed using the test dataset, which contained runs of the experiment separate from those used to train the models. This matched as closely as possible a likely clinical-use scenario, in which the calibration of a device is performed with standard solutions of known concentrations and measurements are performed against this calibration. The models were initially retrained using the whole calibration dataset with the number of factors identified in Table 2 before being used to predict the concentrations of the test dataset. 

**Figure 4:**
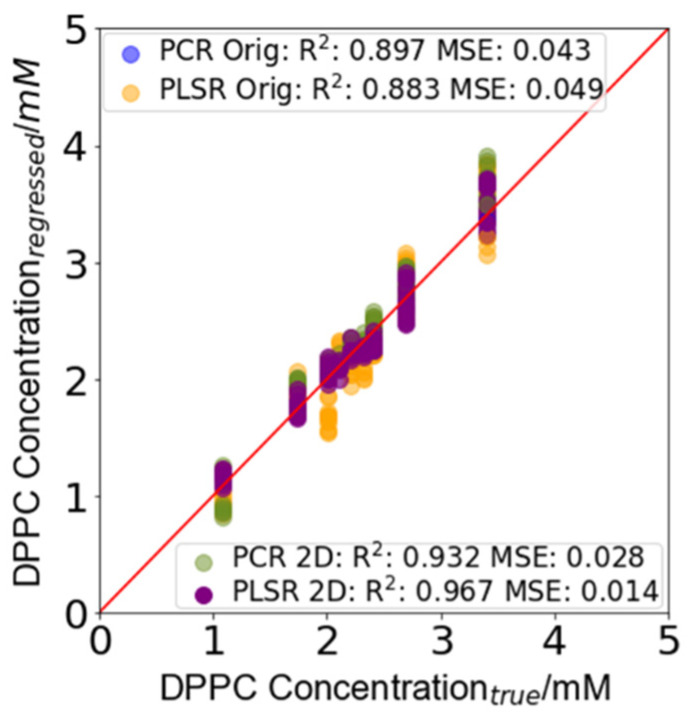
DPPC concentration prediction in the test set samples. The prediction error for each of the models is summarized in Table 3 (further information can be found in Supplementary Information). The largest prediction interval for the PCR Orig model was +0.38 mM, −0.46 m, that for the PLSR Orig model was +0.67 mM, −0.78 m, that for the PCR 2D model was +0.30 mM, −0.38 m, and that for the PLSR 2D model was +0.29 mM, −0.37 mM.

The raw output from each of the models was a concentration for each of the modelled analytes. Figure 4 shows the regressed predictions for each of the models for the sample concentration of DPPC in the test set. A perfect model that predicts the exact concentration would provide an *R*^2^ score of 1 and an *MSE* of 0. In this instance, the SM concentration (not shown) had a perfect score because one concentration was used to train the models. To test this further, samples of L/S ratios at different SM concentrations were used to calibrate the models. The DPPC concentrations in the test set samples were predicted by each of the models and a bootstrapped 95% prediction interval (PI) generated using the calibration set (summarized in Table 3; see also Supplementary Information). The best performing model, with the smallest prediction interval, was the PLSR 2D model (*R*^2^: 0.967, *MSE*: 0.014, *PI*: +0.29 mM, −0.37 mM). Preliminary results, using machine learning models (unreported), showed that the effect of varying SM from 0.0001 to 1 mM could predict SM concentrations within the instrumental error. The worst performance was observed by the PLSR Orig model and 3 LVs (*R*^2^: 0.883, *MSE*: 0.049, *PI*: +0.67 mM, −0.78 mM), and this matched the performance observed in the calibration since the least amount of variance in the calibration data was captured by this model. The PCR Orig model (*R*^2^: 0.897, *MSE*: 0.043, *PI*: +0.38 mM, −0.46 mM) performed better than the PLSR Orig model since more of the variance in the calibration dataset was captured by the model. However, as using a larger number of PCs/LVs also resulted in the greater fitting of the model to noise in the data, the PLSR Orig model did not perform as well as the PCR 2D and PLSR 2D models. The PCR 2D model (*R*^2^: 0.932, *MSE*: 0.028, *PI*: +0.30 mM, −0.38 mM) performed almost as well as the PLSR 2D model and better than both the models based on the original spectra.

To understand how the models would perform if deployed when testing samples at an L/S ratio of 2.2, the mean upper and lower prediction intervals were taken for each model. The mean test set prediction interval using the PCR Orig model for L at a concentration of 2.2 mM was obtained as a concentration interval of 1.76–2.55 mM DPPC. For the same L concentration, the PLSR 2D model provided a prediction interval of 1.86–2.45 mM *DPPC*, which was itself narrower than that of the PCR Orig model by 0.2 mM overall. The performance of the PCR 2D model (1.86–2.49 mM) was better than both the PCR Orig and PLSR Orig models (1.68–2.49 mM), the second of which had the worst overall performance.

### 4.5. L/S Ratio Prediction

**Figure 5:**
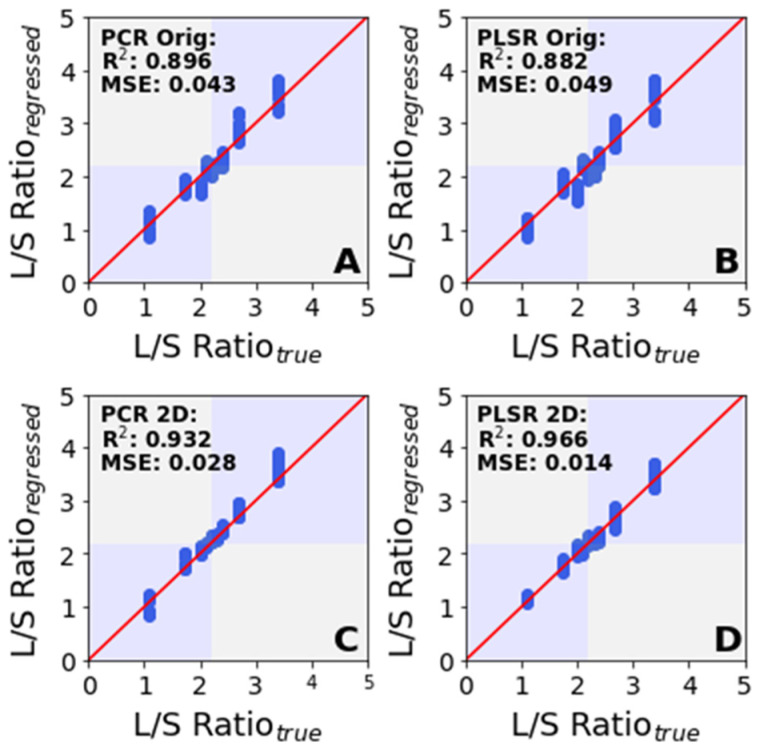
Performance of the regression models in predicting the L/S ratio. The maximum prediction interval range in (**A**) is +0.381, −0.461; in (**B**) is +0.669, −0.777; in (**C**) is +0.302, −0.377; and in (**D**) is +0.291, −0.371.

Figure 5 shows the regression performance for each of the four models (A, B, C and D) summarized in Table 2, where the *R*^2^ and *MSE* was based on the L/S ratio, which was calculated from the predicted constituent L and SM concentrations. Figure 5A,B illustrates predictions based on the original spectra and Figure 5C,D illustrates those based on the second derivative spectra. The best performance was observed with the PLSR 2D model based on 3 LVs (*R*^2^: 0.966, *MSE*: 0.014), with the mean prediction interval for an L/S ratio of 2.2 of 1.86–2.46. The worst performance was observed with the PLSR Orig model (*R*^2^: 0.882, *MSE*: 0.049), with a prediction interval of 1.68–2.74. The PCR 2D model (*R*^2^: 0.932, *MSE*: 0.028), with a prediction interval of 1.68–2.49, performed better than both the PCR Orig (*R*^2^: 0.896, *MSE*: 0.043) model with a PI of 1.76–2.55 and the PLSR Orig model but worse than the PLSR 2D model.

Overall, the models based on the second derivative spectra performed better than those based on the original spectra in spite of the reduction in the signal-to-noise ratio. As these models were generated for L/S ratios based on a 1 mM SM concentration, they would be valid only for similar SM concentrations when deployed. If, however, these models were trained using a larger range of SM concentrations, they could be applied over a larger concentration range. To be applied in a clinical context, a similar characterization would need to be performed using patient samples encompassing the range of expected values. For a point-of-care device, whose primary purpose is to provide a rapid indication of biomarker concentrations, this study indicates that a second derivative model of the spectra would best serve the purpose of providing the required end user information (along with its associated uncertainty) upon which to base the decision to treat for nRDS. 

The calibration process is key to establishing the working concentration range and performance of algorithms. Providing more spectra at relevant concentrations for which the biological samples can be made available will serve to reduce the errors in models. For a point-of-care device deployed in a clinic, such a calibration could prove onerous. The necessary measurement is that of the ratio of a sample spectrum against a background and, as such, is amenable to the use of a calibration library performed using machines with large signal-to-noise ratios. Such libraries could be generated in controlled settings beforehand, allowing for a device in the field to be reconfigured as required. The performance of such devices would be limited only by the signal-to-noise ratio and would provide an end user with a versatile machine able to potentially perform any biomarker measurement based on the mid-IR absorbance. In this manner, it is possible to envisage a point-of-care device able to screen many people in a primary care setting, thus permitting faster access to targeted care.

## 5. Conclusions

This paper demonstrates the determination of the ratio of dipalmitoylphosphatidylcholine (DPPC) and sphingomyelin (SM) in a liquid sample using ATR–FTIR spectroscopy. DPPC and SM are two important biomarkers for neonatal respiratory distress syndrome (nRDS), for which a ratio of less than 2.2 is diagnostic. Using mid-IR spectroscopy, DPPC and SM were quantified and the L/S (DPPC/SM) ratio was determined at a 1 mM SM concentration. Principal component regression (PCR) and partial least square regression (PLSR) were assessed as chemometric approaches using 259 measured spectra of DPPC and SM in dichloromethane over a range of concentrations. Both the raw baseline offset-corrected spectra and second derivative spectra were used in the assessment, and in each case, approximately 60% of the spectra were used for training of the algorithms and the rest were used to validate the algorithms. The PLSR model applied to the second derivative spectra provided the best correlation with known concentrations and allowed for the determination of the L/S ratio close to 2.2 with a prediction interval of 1.86–2.46.

These results pave the way for a point-of-care device used to quantify biomarkers present in liquid clinical samples. However, this conventional ATR–FTIR approach suffers from the weak absorption exhibited by the target analytes that, in turn, limits the minimum detectable concentrations. Using surface enhancement techniques to increase the effective path-length combined with quantum cascade laser (QCL) sources with higher power spectral density will improve signal-to-noise and signal-to-background limitations and enable the development of more compact devices and instrumentation—both of these avenues are currently being explored, and the progress on this will be duly reported.

The presented method is not limited to establishing L/S ratios, as it can be applied to any biological sample with an interpretable mid-IR spectrum, subject to the limit of quantification of the apparatus. In this manner, many other biomarker concentrations and disease states could be assessed at the point of care [32] to usher in a new paradigm in medical diagnosis—to transfer diagnoses to a primary care setting, thus leading to faster patient access to targeted therapy. We suggest alternative calibration approach whereby centrally collated data can develop a standard model for use in deployed devices, serving as an integrity check and informing a potentially longer service/calibration interval.

## Figures and Tables

**Table 1:** Peak Assignments for DPPC and SM.

DPPC Major Peaks	Assignment	SM Major Peaks	Assignment
2957	C-H stretch	2919	C-H stretch
2926	C-H stretch	2851	C-H stretch
2854	C-H stretch	1646	Amide I
1734	C=O stretch	1468	C-H bend
1467	C-H bend	1090	PO2 stretch
1172	C-O stretch	1060	COH stretch
1092	PO2 stretch	967	Choline stretch
1065	C-O stretch of phosphodiester		
966	Choline stretch		

**Table 2:** Model summary (all values to 3 sig. figs.).

Model	PCs/LVs	*R* ^2^ *CV*	*MSE CV*
PCR Orig Model	5	0.989	0.006
PLSR Orig Model	3	0.976	0.013
PCR 2nd derivative (2D) Model	3	0.980	0.010
PLSR 2nd derivative (2D) Model	3	0.985	0.008

**Table 3:** Summary of the maximum and minimum prediction intervals for each model. The max interval range indicates the maximum range of the prediction interval for the point with the largest prediction interval within the test set.

Model	Maximum Positive Interval (mM)	Maximum Negative Interval (mM)	Max Interval Range (mM)
PCR Orig	0.38	0.46	0.85
PLSR Orig	0.67	0.78	1.45
PCR 2D	0.30	0.38	0.68
PLSR 2D	0.29	0.37	0.67

## Data Availability

All data supporting this study are available from the University of Southampton repository at: https://doi.org/10.5258/SOTON/D1829 (accessed on 25 January 2022).

## Supplementary Materials

The following supporting information can be downloaded at: https://www.mdpi.com/article/10.3390/s22051744/s1, Figure S1: Prediction intervals for predictions of the DPPC concentration made by the PCR Orig model for test set data; Figure S2: Prediction intervals for predictions of the DPPC concentration made by the PLSR Orig model for test set data; Figure S3: Prediction intervals for predictions of the DPPC concentration made by the PCR 2D model for test set data; Figure S4: Prediction intervals for predictions of the DPPC concentration made by the PLSR 2D model for test set data; Figure S5: Prediction intervals for predictions made by all the models for the L/S ratio of the test set samples; Figure S6: Data processing pathway showing the data flow for both the test and training datasets to generate a calibration model for prediction of the test set L/S ratios and their associated prediction intervals.

## Abbreviations

SM	*N*-Palmitoyl-D-erythro-sphingosylphosphorylcholine
DPPC	1,2-dipalmitoyl-sn-glycero-3-phosphocholine
nRDS	Neonatal respiratory distress syndrome
PCR	Principal component regression
PLSR	Partial least squares regression
L/S	Lecithin/sphingomyelin ratio
ATR–FTIR	Attenuated total reflectance–Fourier transform infrared spectrometry
MSE	Mean squared error
*R* ^2^	Coefficient of determination
RMSE	Root mean squared error
PI	Prediction interval
PCs	Principal components (model factors for principal components regression)
LVs	Latent variables (model factors for partial least squares regression)
AF	Amniotic fluid
BAL	Bronchoalveolar lavage
GA	Gastric aspirate
