# Supplementary material for: Prediction of Neonatal Respiratory Distress Biomarker Concentration by Application of Machine Learning to Mid-Infrared Spectra
Source: Sensors (Basel). 2022 Feb 23;22(5):1744. doi: 10.3390/s22051744 (PMC8914945; doi:10.3390/s22051744)
Supplement: Supplementary file 1 [file sensors-22-01744-s001.zip › sensors-1589289-supplementary.pdf]

Supplementary Information

This supplementary information contains further graphs showing the prediction intervals around each prediction made by the models on the test set using the bootstrap method referenced in the main text.

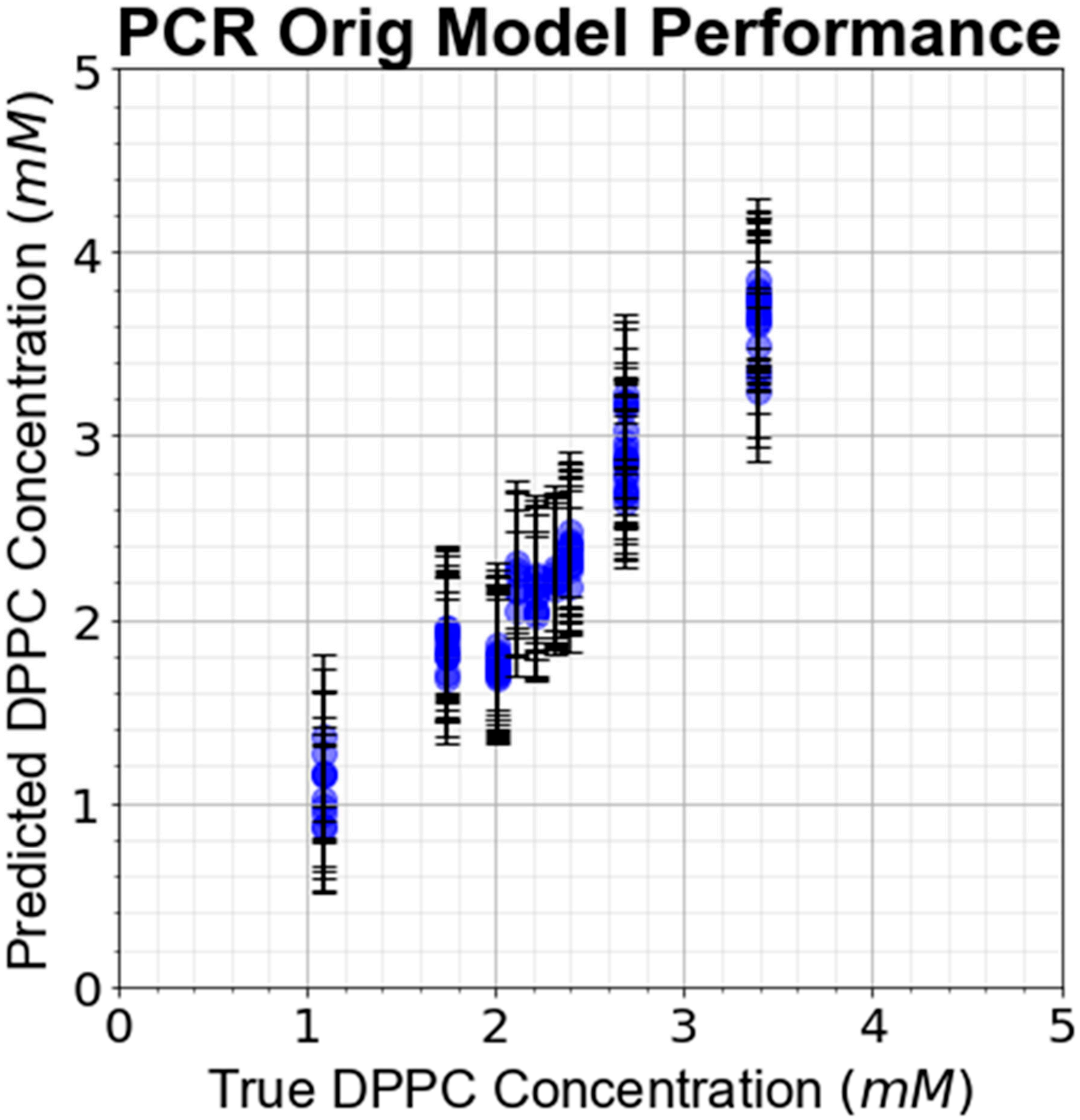

Figure S1. Prediction intervals for predictions of the DPPC concentration made by the PCR Orig model for test set data.

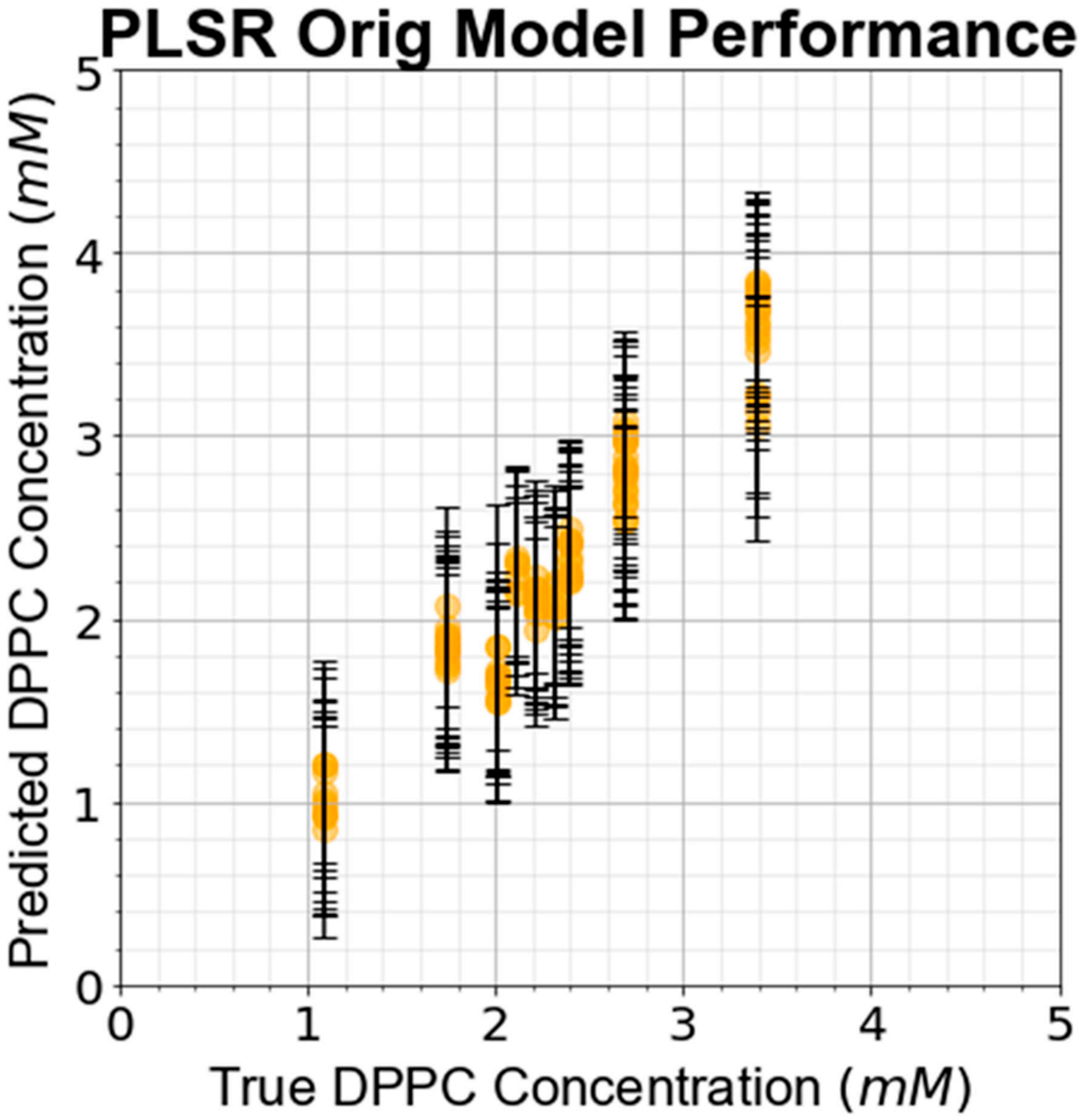

Figure S2. Prediction intervals for predictions of the DPPC concentration made by the PLSR Orig model for test set data.

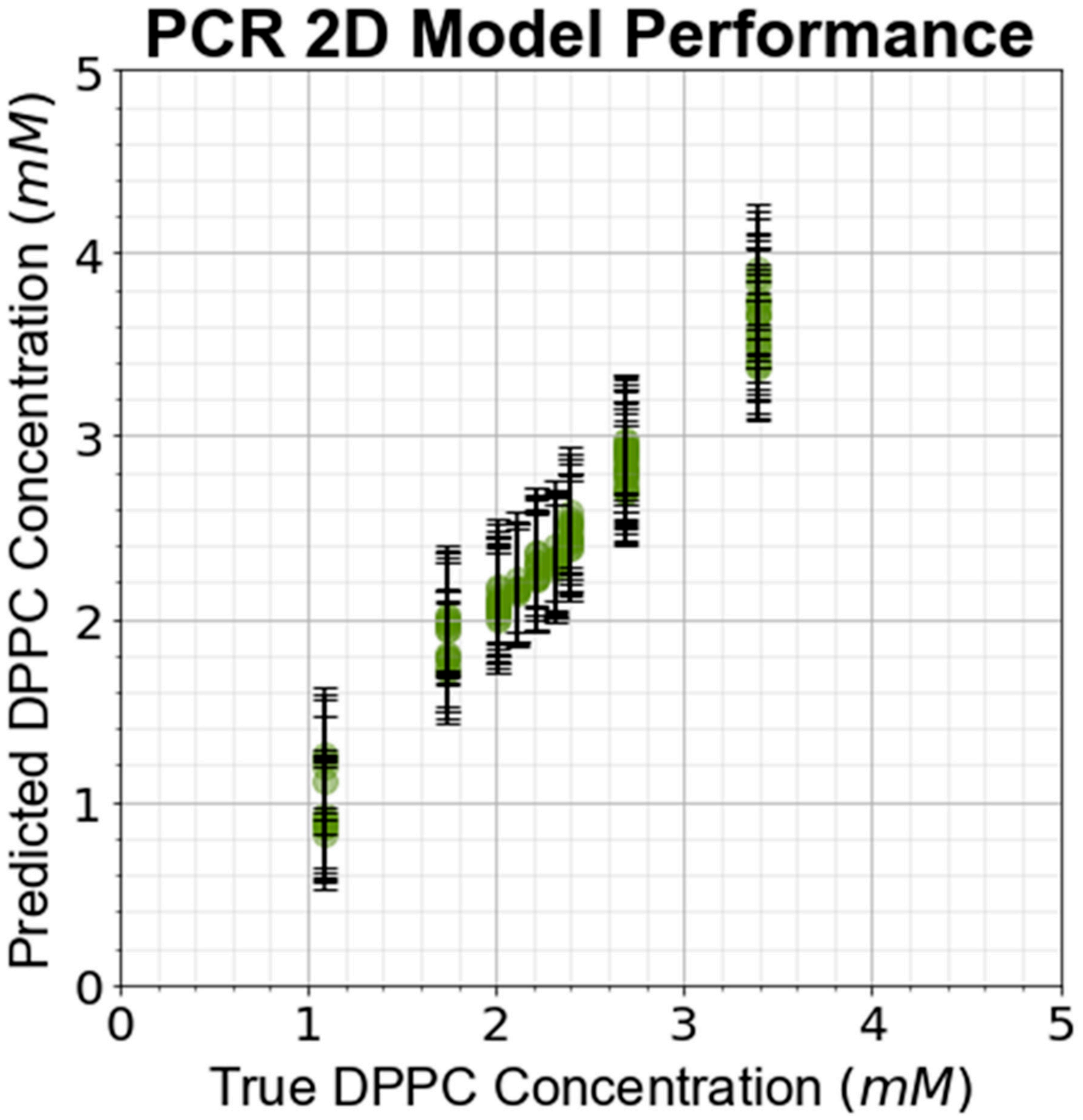

Figure S3. Prediction intervals for predictions of the DPPC concentration made by the PCR 2D model for test set data.

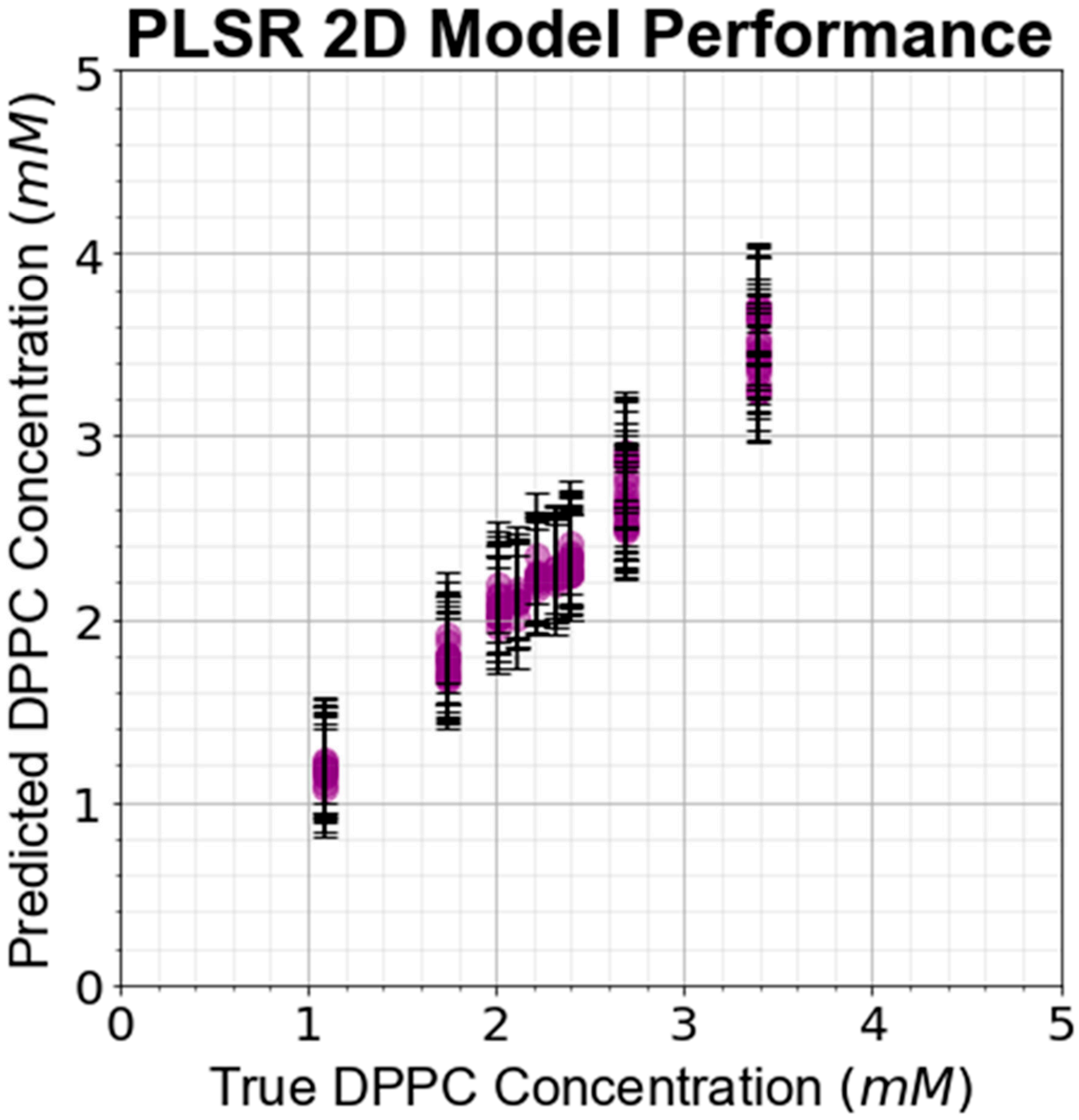

Figure S4. Prediction intervals for predictions of the DPPC concentration made by the PLSR 2D model for test set data.

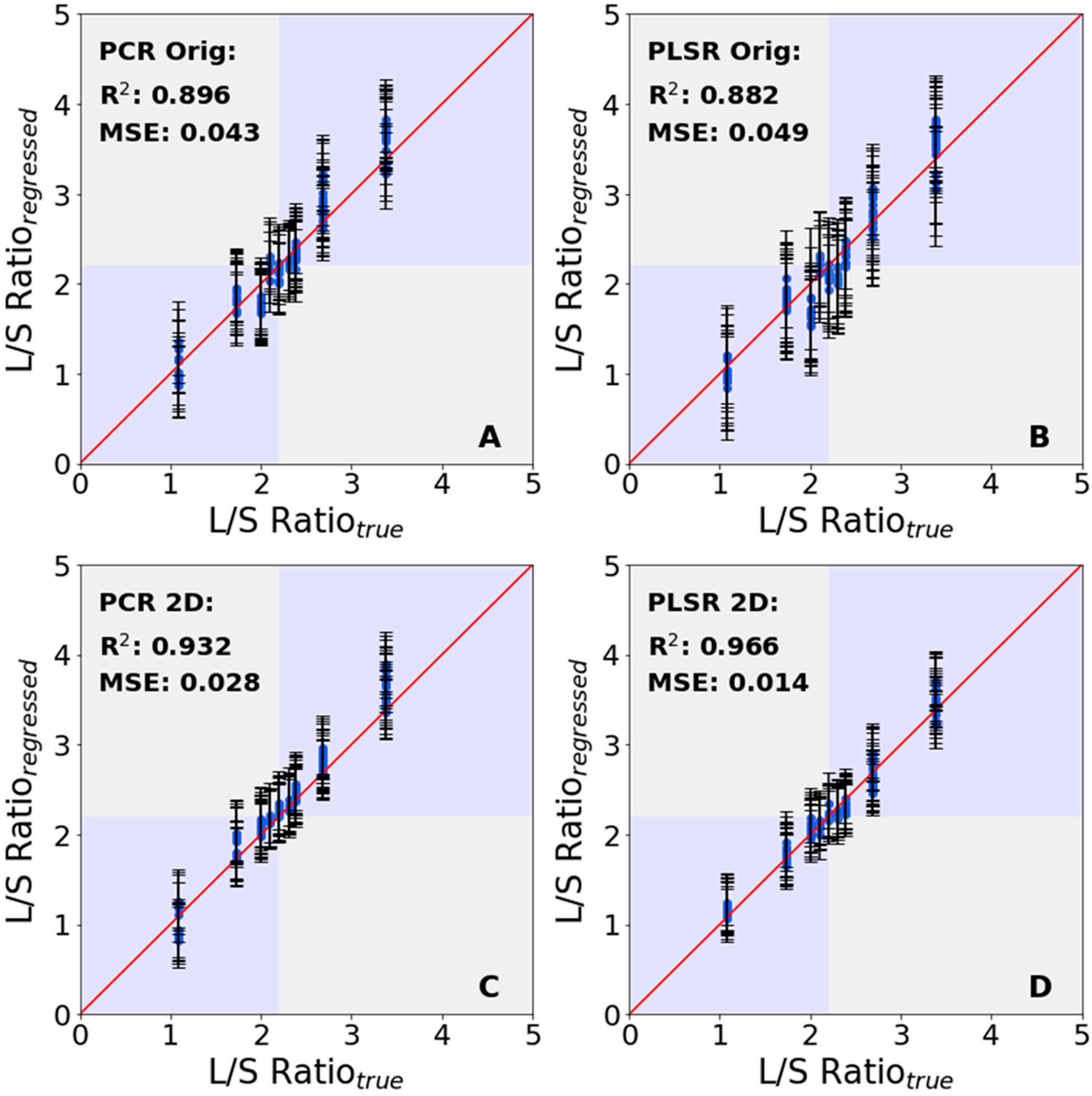

Figure S5. Prediction intervals for predictions made by all the models for the L/S ratio of the test set samples

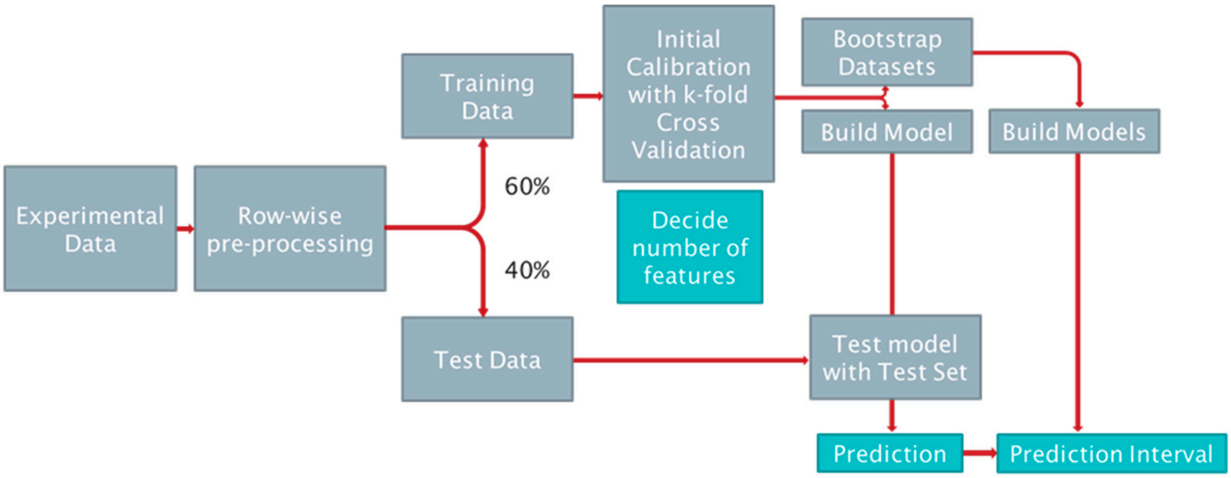

Figure S6. Data processing pathway showing the data flow for both the test and training datasets to generate a calibration model for prediction of the test set L/S ratios and their associated prediction intervals.
